# Comment on "Lidocaine and pinacidil added to blood versus crystalloid cardioplegic solutions: study in isolated hearts"

**DOI:** 10.21470/1678-9741-2018-0302

**Published:** 2018

**Authors:** Claus J. Preusse

**Affiliations:** 1 Department of Cardiac Surgery, University Bonn, Bonn, Germany

In the issue 33(3) of the Brazilian Journal of Cardiovascular Surgery, Carmo et al.^[1]^ published a paper entitled "Lidocaine and pinacidil added to blood *versus* crystalloid cardioplegic solutions: study in isolated hearts". The authors report on studies in rat hearts using a Langendorff model. Besides a control group, two other groups were investigated: Custodiol HTK solution and Del Nido solution. Pinacidil was added to both solutions, while lidocaine was only added to Custodiol, since Lidocaine is already an essential compound of Del Nido solution. After 180-min-ischemia at 4ºC all hearts were aerobically reperfused for 90 mins and the following parameters were analyzed (unfortunately only as percentage of recovery): contractility, coronary resistance and alpha-fodrin degeneration.

The authors reported on superior outcome by Del Nido solution after 90-min-reperfusion.

Although the study shows some interesting approaches to intraoperative myocardial protection, some aspects are objectionable regarding their comparative analytical methodology.

First, only one compound - pinacidil - was added to Del Nido solution, while two, pinacidil and lidocaine, were added to custodiol, since lidocaine is already an essential component of Del Nido solution. This is not consistent with a clean, scientifically acceptable comparative analysis, as Del Nido already meets one of the parameters subject to the investigation.

Second, to measure myocardial contractility, mostly dP/dt max. is used in appropriate experimental or clinical investigations. However, from scientific point of view this is not completely accurate, since the time until the maximal pressure is reached, may play an important role. Therefore, it would be correct to additionally divide dP/dt by t (time).

Third, Custodiol is the only highly buffered cardioplegic solution available worldwide. The efficacy of buffers in any cardioplegic solution can be clearly demonstrated by simultaneous measurements of extracellular myocardial pH and of myocardial lactate content during ischemia^[2]^. Lactate/pH relationships differ depending on the cardioplegic solutions applied and concerning a buffered solution, the lactate/pH relationship depends on the nature of the buffer applied, on buffer concentration and, on additional compounds in a cardioplegic solution^[3]^. Any drug that stabilizes myocardial membranes, will impair the permeation of H^+^-ions from the intra- to the extracellular space. Such impaired permeation leads to an intracellular 'overload' of H^+^-ions and, conversely, to less acidification of the myocardial extracellular space. Consequently, the lactate/pH curve will shift towards alkalosis! This effect should occur if procaine or lidocaine are parts of the solution, since both drugs are membrane stabilizing drugs. In experiments on ischemic hearts of mongrel dogs at 15ºC we compared histidine buffered solutions with and without procaine (Figure 1). In this experiment we were able to demonstrate the aforementioned effect on the extracellular pH. Although both solutions contained equal buffer capacities, the extracellular pH is more alkalotic in the procaine group. This means that the total available extracellular buffering capacity is not effectively utilized, *i.e*. one of the principal benefits of the solution - its buffering capacity - is not sufficiently realized^[4]^. Therefore, it is not surprising that the custodiol-LP group led to inferior results.

**Fig. 1:**
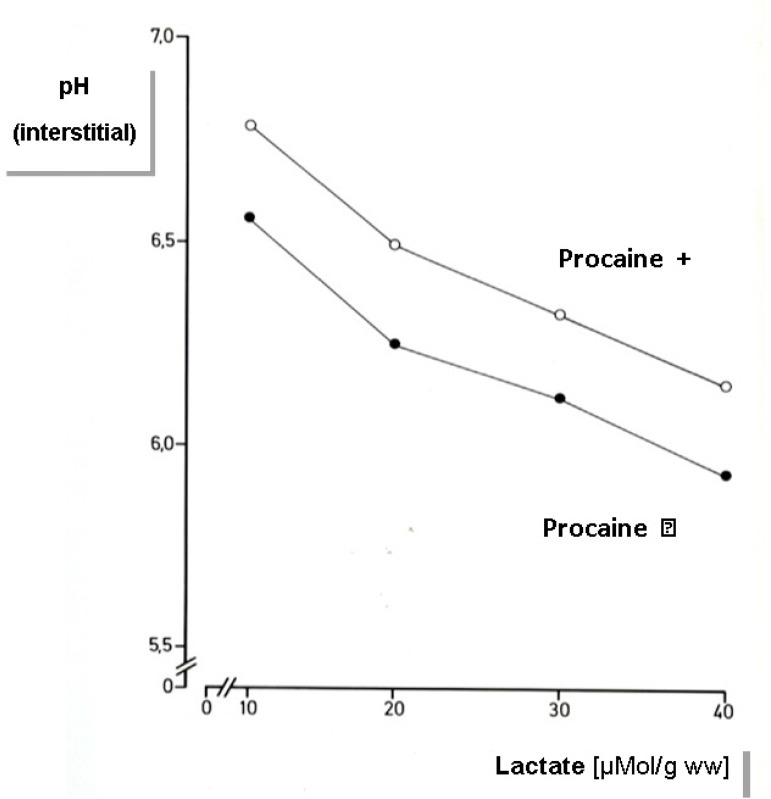
Experiments were performed on ischemic mongrel dog hearts at 15ºC. For myocardial protection Custodiol solution with or without procaine was used. (Methods see also Preusse et al.^[2]^)

The results of the current study differ from an experimental study in South Korea in 2003 which was also performed on rat hearts (!) being protected either by original custodiol or by Del Nido solution. It became obvious that after two-hour-arrest mitochondrial scoring was superior in the custodiol group^[5]^.

Given the aforementioned objections, we would question the conclusions drawn by the authors. They would have been correct, had they concluded that a decisively modified (!) Custodiol solution - mainly by lidocaine - causes inferior efficacy in myocardial protection due to the relatively reduced buffering power. But their study does not address the intraoperative protective efficacy of the original version of custodiol and therefore, the results are not applicable to clinical usage. In conclusion, this paper does not represent a valid comparative study between custodiol and Del Nido solutions.

## References

[1] Carmo HP, Reichert K, Carvalho DD, Silveira-Filho LM, Vilarinho K, Oliveira P (2018). Lidocaine and pinacidil added to blood versus crystalloid
cardioplegic solutions: study in isolated hearts. Braz J Cardiovasc Surg.

[2] Preusse CJ, Gebhard MM, Bretschneider HJ (1982). Interstitial pH value in the myocardium as indicator of ischemic
stress of cardioplegically arrested hearts. Bas Res Cardiol.

[3] Preusse CJ (2016). Custodiol cardioplegia: a single-dose hyperpolarizing
solution. J Extra Corpor Technol.

[4] Kresh JY, Nastala C, Bianchi PC, Goldman SM, Brockman SK (1987). The relative buffering power of cardioplegic
solutions. J Thorac Cardiovasc Surg.

[5] Kong JH, Kim DH, Chang BH (2003). Comparison of cardioprotection between
histidine-tryptophan-ketoglutarate cardioplegia and DelNido cardioplegia in
isolated rat hearts. Korean J Thorac Cardiovasc Surg.

